# Impact of deep learning‐based multiorgan segmentation methods on patient‐specific internal dosimetry in PET/CT imaging: A comparative study

**DOI:** 10.1002/acm2.14254

**Published:** 2024-01-12

**Authors:** Mehrnoosh Karimipourfard, Sedigheh Sina, Hojjat Mahani, Mehrosadat Alavi, Mehran Yazdi

**Affiliations:** ^1^ Department of Ray‐Medical Engineering Shiraz University Shiraz Iran; ^2^ Radiation Research Center Shiraz University Shiraz Iran; ^3^ Radiation Applications Research School Nuclear Science and Technology Research Institute Tehran Iran; ^4^ Department of Nuclear Medicine Shiraz University of Medical Sciences Shiraz Iran; ^5^ School of Electrical and Computer Engineering Shiraz University Shiraz Iran

**Keywords:** deep learning, internal dosimetry, PET/CT, segmentation

## Abstract

**Purpose:**

Accurate and fast multiorgan segmentation is essential in image‐based internal dosimetry in nuclear medicine. While conventional manual PET image segmentation is widely used, it suffers from both being time‐consuming as well as subject to human error. This study exploited 2D and 3D deep learning (DL) models. Key organs in the trunk of the body were segmented and then used as a reference for networks.

**Methods:**

The pre‐trained p2p‐U‐Net‐GAN and HighRes3D architectures were fine‐tuned with PET‐only images as inputs. Additionally, the HighRes3D model was alternatively trained with PET/CT images. Evaluation metrics such as sensitivity (SEN), specificity (SPC), intersection over union (IoU), and Dice scores were considered to assess the performance of the networks. The impact of DL‐assisted PET image segmentation methods was further assessed using the Monte Carlo (MC)‐derived S‐values to be used for internal dosimetry.

**Results:**

A fair comparison with manual low‐dose CT‐aided segmentation of the PET images was also conducted. Although both 2D and 3D models performed well, the HighRes3D offers superior performance with Dice scores higher than 0.90. Key evaluation metrics such as SEN, SPC, and IoU vary between 0.89–0.93, 0.98–0.99, and 0.87–0.89 intervals, respectively, indicating the encouraging performance of the models. The percentage differences between the manual and DL segmentation methods in the calculated S‐values varied between 0.1% and 6% with a maximum attributed to the stomach.

**Conclusion:**

The findings prove while the incorporation of anatomical information provided by the CT data offers superior performance in terms of Dice score, the performance of HighRes3D remains comparable without the extra CT channel. It is concluded that both proposed DL‐based methods provide automated and fast segmentation of whole‐body PET/CT images with promising evaluation metrics. Between them, the HighRes3D is more pronounced by providing better performance and can therefore be the method of choice for 18F‐FDG‐PET image segmentation.

## 1 INTRODUCTION

In the era of precision medicine, positron emission tomography (PET) imaging is a vital technique for the acquisition of molecular data, while suffering from inaccurate anatomical information.^1^, ^2^ The lack of high‐resolution structural data and detailed organ localization are challenging in PET imaging, particularly 18F‐FDG scans.^3^ The challenges have been properly approached by introducing multimodality imaging such as PET/computed tomography (CT) and PET/magnetic resonance (MR) imaging.^4^

Image segmentation has found various applications in computer vision and has risen in popularity all over the globe. (Multi)organ segmentation is crucial in Monte Carlo (MC)‐based internal/external dosimetry using an anthropomorphic phantom to calculate average organ dose and so‐called organ‐specific S‐values in radionuclide imaging and therapy.^5^ Moreover, in radiotherapy treatment planning, accurate delineation of target organs is commonly performed by an expert radiologist and/or radiation oncologist in order to maximize the dose delivered to the tumor and simultaneously minimize the radiation burden to the organs at risk (OARs).^6^ In such tasks, every millimeter of the organs of interest must be accurately segmented.^7^

Conventionally, this segmentation is based on patients’ CT scans and recently on MR images. Albeit, CT‐based treatment planning is hindered by the lack of tumor growth and burden, particularly for early‐stage patients when the tumor is relatively small in size.^8^ Since molecular images provide physiological information about the patients, PET‐based treatment planning has been, therefore, proposed to address the problem. Currently, PET image segmentation is manually performed by an expert radiologist/radiation oncologist to determine target organs. Manual PET image segmentation is subject to several challenges such as lack of accuracy, being time‐consuming nature, and inter‐radiologist variations.^9^

Many studies have investigated semi‐ and fully‐automated PET image segmentation for various applications. Atlas‐based methods are straightforward and show effectiveness for PET image segmentation and are commercially available in some devices.^10^ The limitation of atlas‐based approaches is segmentation accuracy which depends highly on the image registration procedure owing to organ morphology. Also, image artifacts and the unpredictability of tumor shape decrease the performance of the atlas‐based models. In addition to atlas‐based algorithms, DL‐based ones have shown promising performances enabling accurate and fast segmentation of molecular images^11^, ^12^ each with a variety of architectures, hyperparameters, and/or organs of interest.

Deep learning (DL) has gained increasing interest in internal dosimetry^12^ and has found several applications including direct dose estimation^13^ and automatic segmentation of both structural and functional images. Long et al proposed a fully connected network (FCN) architecture in image segmentation tasks enabling end‐to‐end training and pixel‐to‐pixel translation.^14^ In another study, Ronneberger et al. developed a U‐Net based on FCN and then obtained more structural information from up and down sampling layers. These methods were merged with the generative adversarial network (GAN) architecture to boost the performance and generate a synthetic sample with high similarity to the real one. After a while, the p2p‐U‐Net‐GAN was applied to medical tasks.^15^ Dong et al., for the first time, presented a study on automatic multiorgan segmentation in thorax CT with a pixel‐to‐pixel translation U‐Net‐GAN architecture for lung cancer patients.^16^ Their proposed method could delineate the left and right lungs, spinal cord, esophagus, and heart on chest CT images for 35 patients. Their obtained Dice similarity coefficient varied between 0.75 and 0.97, and the mean surface distance of the five OARs showed the encouraging performance of the network. In another study, Chen et al focused on cervical tumor segmentation in 18F‐FDG PET images mostly because of its difficulty due to proximity with the bladder both with a high amount of 18F‐FDG uptake.^9^ They proposed a supervised machine learning method with convolutional layers to segment cervical tumors. They mapped the PET images to their corresponding label maps, in which the organs were labeled as −1, 0, and 1. Their prior information constraint spatial information embedded CNN (PIS‐S‐CNN) method was evaluated with a mean Dice similarity coefficient of 0.84 and then was compared with U‐Net, FCN‐8 stride, and FCN‐2 stride architectures. Their proposed network yielded accurate results for segmenting cervical tumors. Bourigault et al published a study on PET/CT tumor segmentation using a full‐scale U‐Net network.^17^ They trained multiple neural networks for tumor volume segmentation and achieved an average Dice coefficient of 0.75.

While there are several works on the utilization of the DL for either specific organ segmentation in PET images or direct internal dose estimation, the literature is still premature and mandates the investigation of new DL networks for simultaneously multiorgan segmentation of functional images as a key step in 3D image‐based internal dosimetry tasks. To this end, this study aims at exploiting pix‐to‐pix GAN, first proposed by Dong et al, for fully automatic, accurate, and high‐resolution multiorgan PET‐only image segmentation. A fair comparison is also conducted with the well‐known HighRes3D, first proposed by Li et al.^16^, ^18^ considering two deep networks: PET‐only‐based as well as PET/CT‐based trained models (HighRes3DPET and HighRes3DPET/CT, respectively) to further investigate the performance of the network with and without the inclusion of anatomical CT data as an extra input channel. The HighRes3D network is implemented in the NiftyNet open‐source platform. The main contribution of the present study is, therefore, the utilization of the pix‐to‐pix GAN for PET/CT image segmentation. Furthermore, the DL‐assisted estimation of several organs’ volumes and more importantly the impact of the DL‐based segmentation methods on the calculated S‐value of key organs, as a link between deep PET image segmentation and internal dosimetry in diagnostic nuclear medicine, have been also studied. Several evaluation metrics including the sensitivity (SEN), specificity (SPC), intersection over union (IoU), and Dice coefficient would be calculated and compared.

## 2 MATERIALS AND METHODS

**FIGURE 1:**
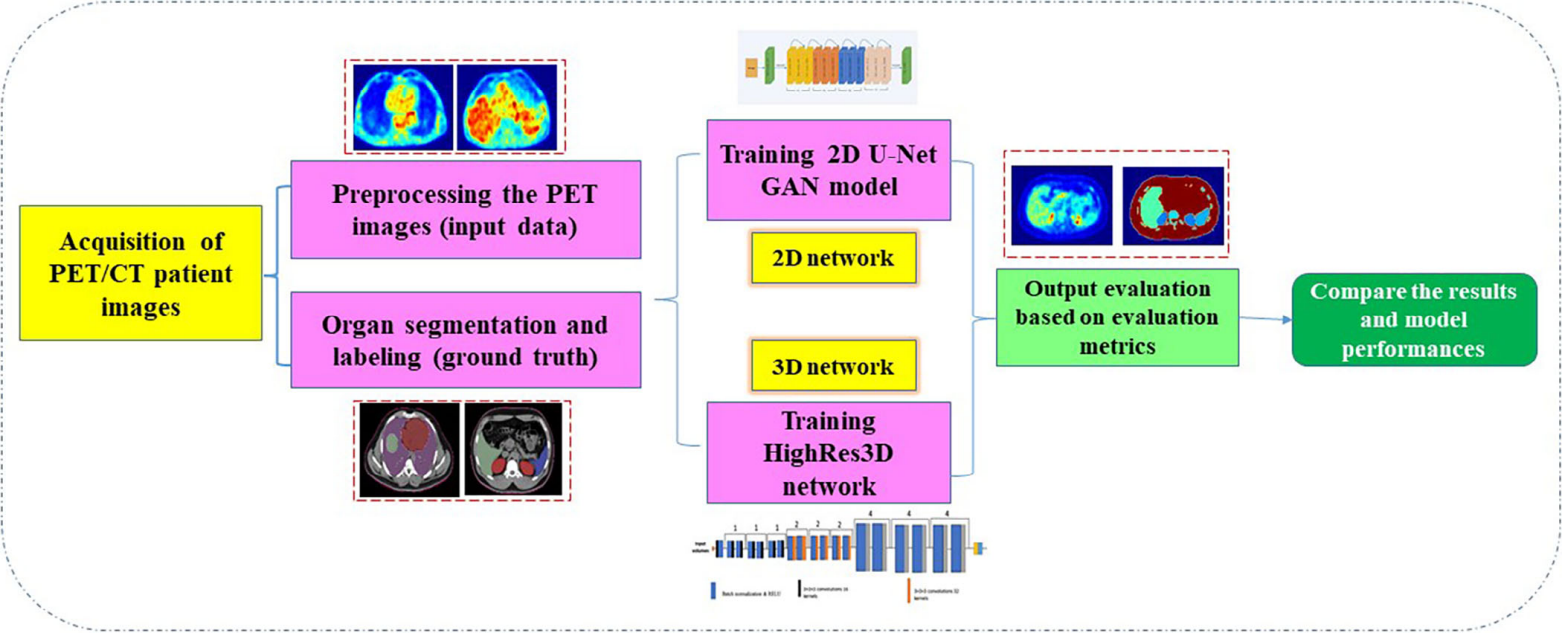
General workflow of the present study to compare 2D U‐Net GAN and HighRes3D_PET_ for PET image segmentation.

In the present study, we exploited DL methods in 2D and 3D setups to segment patient organs undergoing PET/CT imaging. Multiorgan segmentation based on the PET images is one of the challenging problems in dosimetry tasks which can be automatically and accurately facilitated with the DL networks. Figure 1 shows the general workflow of this work.

### 2.1 Data acquisition and dataset

**FIGURE 2:**
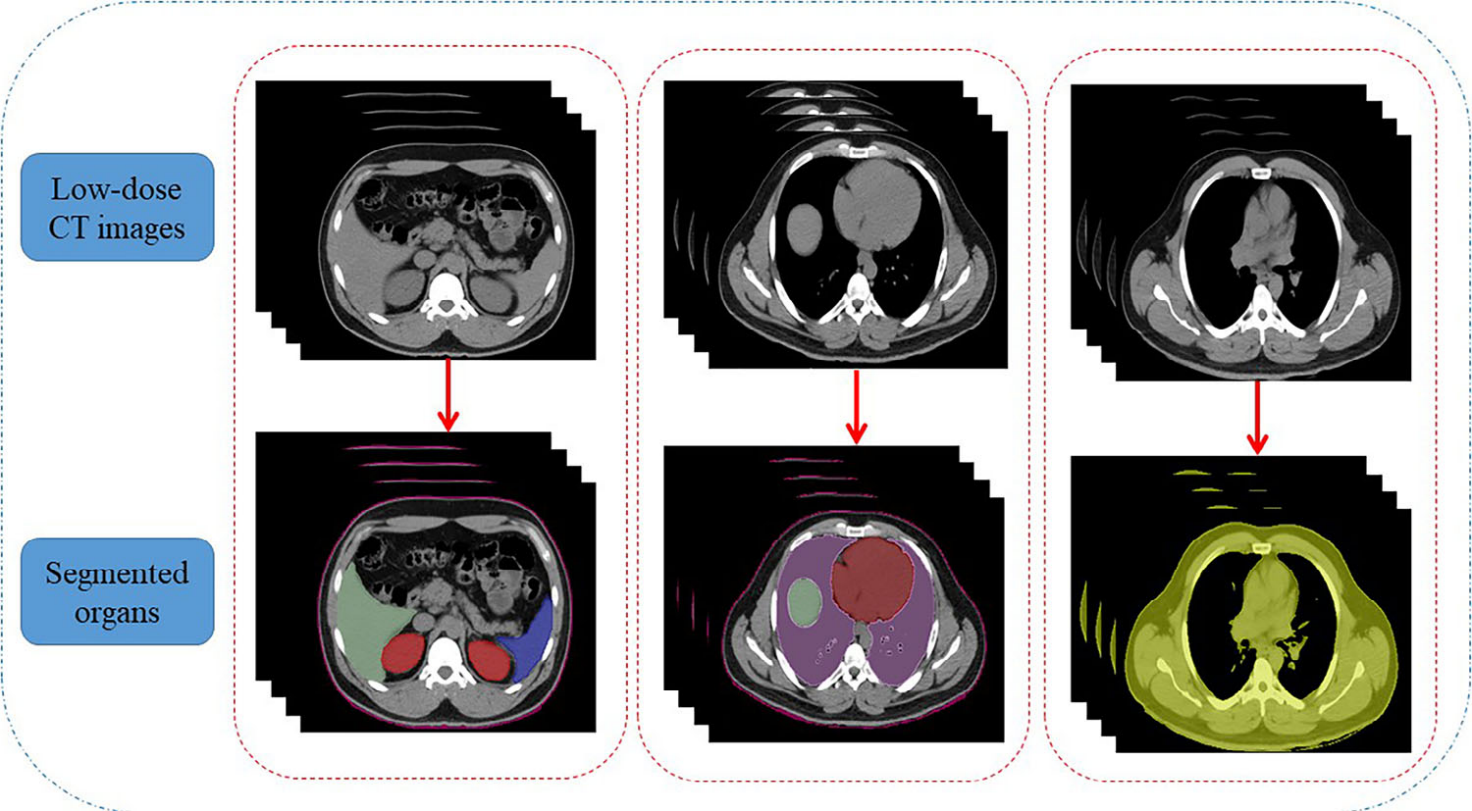
The segmented organs using thresholding, region growing, and level tracing methods.

Whole‐body unenhanced 18F‐FDG PET‐CT images of 50 patients were acquired using a PHILIPS PET/CT scanner, series Ingenuity TF 64 slices with LYSO crystals, and 18 cm axial field of view (FOV). The rights for access to data were approved by Shiraz University (Ethical code: IR.US.REC.1401.020). The scanning process lasted between 30 and 300 s per bed, and the scans were performed at 60‐min post‐injection. The PET attenuation‐corrected images had a 144 × 144 matrix size with 4 × 4 × 4 mm^3^ voxel size, and the CT images had dimensions of 512 × 512 × 320. To prepare a registered and fully aligned training dataset, all PET slices of the trunk and their corresponding low‐dose CT images were translated. Afterward, the low‐dose CT images were resampled to match the PET images. Owing to the resolution limitations of PET images, three preprocessing procedures were applied^1^: denoising using a median filter with neighborhood size of 1 × 1 × 1 (thresholding at the 20‐percentile level to boost SNR) and the smoothing process using Gaussian kernel with 2 mm sigma for each frame,^2^, ^19^ normalization into [0 1] scale, and^3^ resampling to a 256 × 256 grid. The regions of interest (ROIs) were delineated on the axial low‐dose CT images for seven organs and two regions: bladder, kidney, stomach, liver, lung, spleen, heart wall, body contour, and bone skeleton regions, respectively. The ROIs were drawn manually using thresholding, region growing, and level tracing in 3D Slicer software version 4.8.1. Figure 2 shows the segmented organs related to the low‐dose CT images.

**FIGURE 3:**
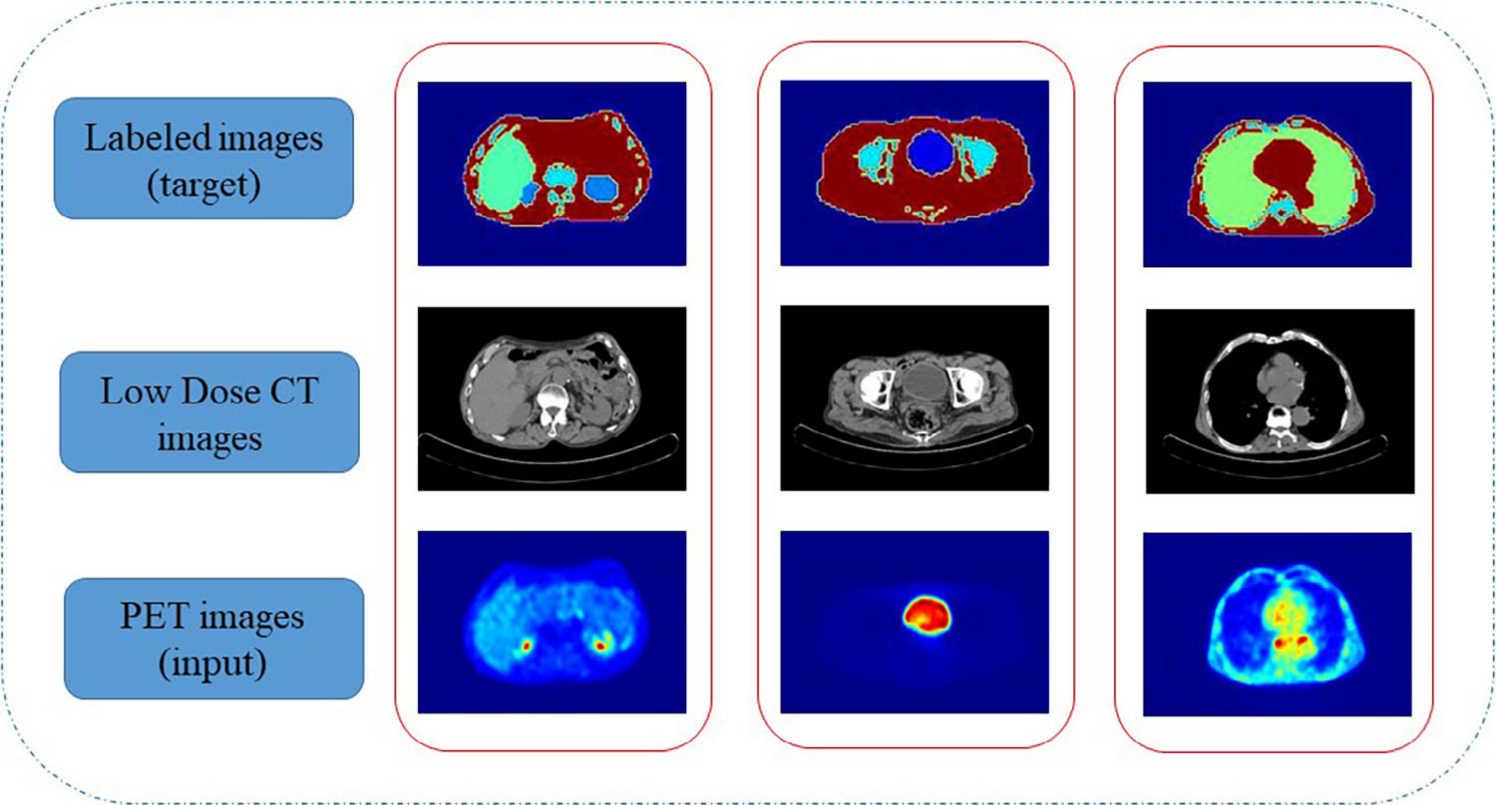
The paring of input (PET images) with output (labeled images) for both 2D and 3D networks. The middle row represents the corresponding low‐dose CT images.

Each axial slice required further manual deformation of the active contours to completely outline the organs. Segmented organs were labeled in each slice and paired with the corresponding PET and low‐dose CT images (see Figure 3). In Figure 3, the labeled images (top row) are produced by manual segmentation of the corresponding low‐dose CT images (middle row). The paired images were categorized into 2D and 3D cohorts for 2D and 3D networks, respectively. The ground truth and organ delineation were defined by an expert nuclear medicine physicist and then confirmed by a nuclear medicine specialist. While the dataset consists of grayscale images, the organs were color‐coded for a better representation of the label maps illustrated in Figure 3.

The network training was performed in 2D and 3D fashions. For the 2D approach, raw PET images were used as input for a p2p‐U‐Net‐GAN network to predict the segmented images. The pretrained p2p‐U‐Net‐GAN was weighted using the Facades dataset with 2D convolutional layers.^20^ In this study, 5000 parried PET images and segmented slices were prepared. Seventy percent, 15%, and 15% of the data were partitioned into the training, validation, and test sets, respectively. Fifty patient cases were enrolled for 3D network training, validation, and testing.

### 2.2 DL preliminaries

The principle of the GAN framework consists of two independent networks, a generator, and a discriminator, in which each network is carried out by a specific function and set of parameters.^21^ Generator *G* aims to generate fake but plausible images that begin their function using the random data distribution in latent spaces, whereas discriminator *D* is responsible for distinguishing the real and forged images. The convergence of the two networks is achieved by reaching Nash equilibrium based on game theory.^20^ Based on the success of conditional GANs (cGANs), the generator network is replaced with a conditional variant to adapt adversarial networks from image generation to translational tasks. In this approach, the translation is mapped between two domains, a source domain image, and a corresponding ground truth target image, via the mapping function. An example in the medical field is the translation of noisy PET images to the corresponding denoised ones.^22^ The pix‐to‐pix model is the underlying principle of cGAN that operates in the image‐to‐image translation area. In other words, pix‐to‐pix implementation is a generalized framework for GANs. In this case, the adversarial loss plays a significant role in training and encouraging the generator to generate plausible images via the target domain.^20^

#### 2.2.1 2D network setup

**FIGURE 4:**
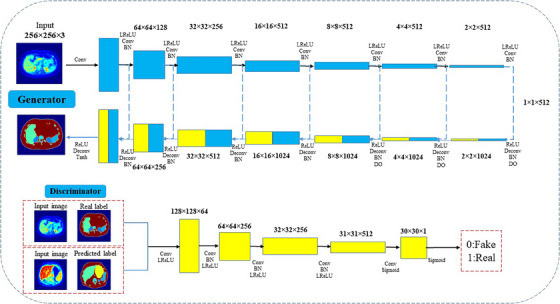
Sketch of the p2p‐U‐Net‐GAN used in the present study to segment PET/CT images.

The discriminator model performs as a typical classification FCN with several 2D‐convolution and leaky‐ReLU functions, which are optimized using binary cross entropy and the Adam optimizer. The hyperparameter arrangements are listed in Table 1. The generator model functions as an encoder‐decoder using U‐Net design. The source images were inserted as inputs, and during the down‐sampling and up‐sampling processes, the target images were generated as outputs. Skip connections are between down‐sampling and up‐sampling layers that form a U‐shape. The generator layers were convolutional, batch normalized, and dropout layers, and the tanh function were used in the output layer. The generator model is trained based on the discriminator, which is functioned to minimize the predicted loss from the discriminator and L1 loss and is finally updated via a weighted sum of both the adversarial and L1 losses. Figure 4 shows the p2p‐GAN architecture and layers. The generator and discriminator were which converged after 200 epochs in network training. It should be highlighted that the p2p‐GAN was trained only with PET images. To segment the entire PET dataset, all 2D PET slices are then sequentially fed into the p2p2‐GAN model (i.e., slice‐by‐slice).

**TABLE 1:** The hyperparameters of p2p‐U‐Net‐GAN.

Mini batch sizes	D learn rate	D beta 1	D beta 2	G learn rate	G beta 1	G beta 2
22	0.01	0.8	0.89	0.001	0.9	0.89

#### 2.2.2 3D network setup

**FIGURE 5:**
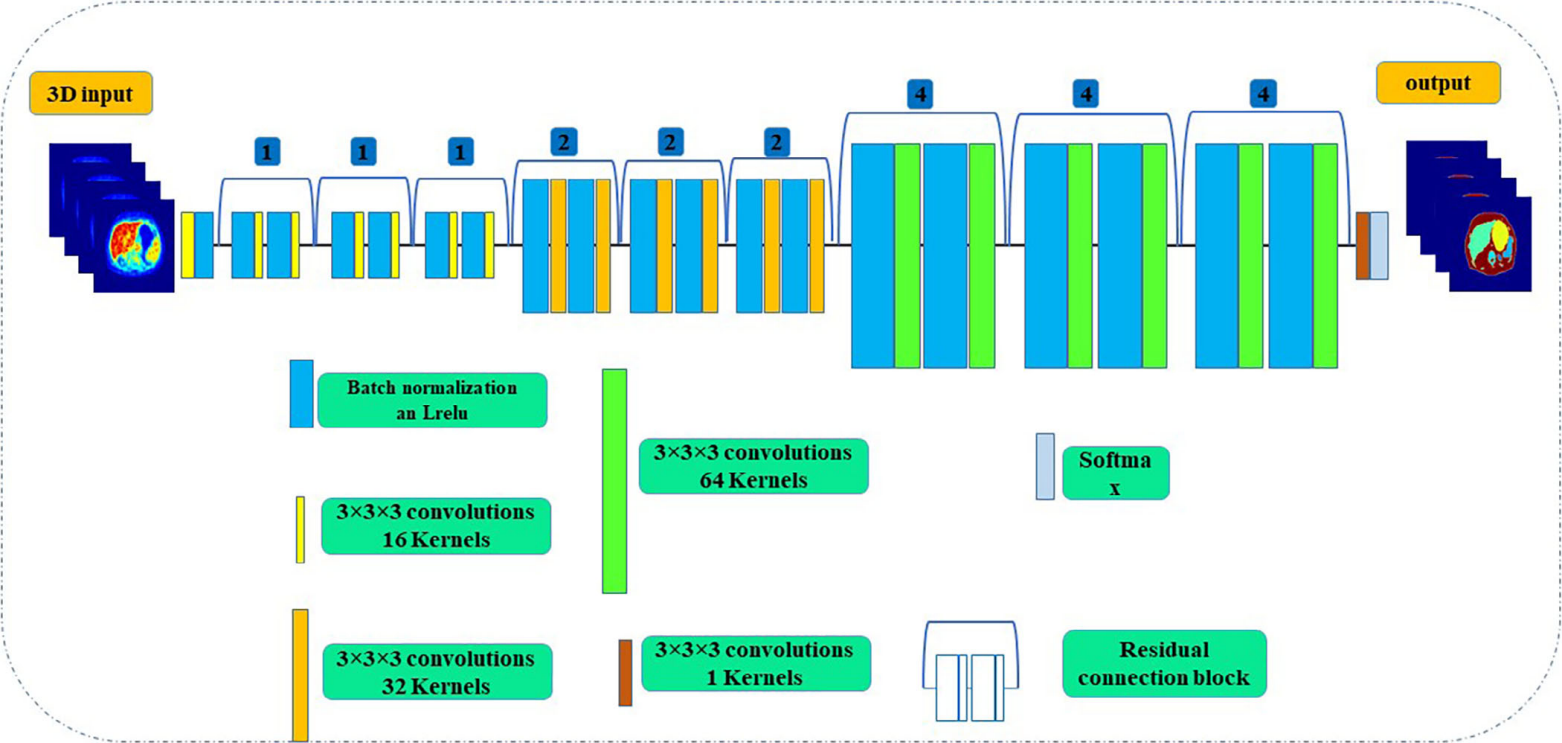
Sketch of the HighRes3D_PET_ architecture used in the present study to segment PET images.

A ResNet model was employed for the prediction of segmented images using the NiftyNet platform, an open‐source TensorFlow‐based platform specialized for medical image analysis. The NiftyNet platform provides tools for image segmentation and facilitates training in the medical imaging field.^23^ The HighRes3DPET, as exhibited in Figure 5, includes 20 convolutional layers, and each residual block consists of a batch normalization layer and Leaky‐ReLU pre‐activation order. The configuration file was set with a batch size of 20, Adam optimizer, and 0.001–0.01 changing learning rate. One hundred validation procedures were tuned after 10 epochs, and a total of 200 epochs were adjusted to run. A similar model was also exploited for the HighRes3DPET/CT except for an extra low‐dose CT input channel.

The present study was conducted using the Deep Learning toolbox of MATLAB 2021a software and also the NiftyNet platform all on a computational system with Windows 10 OS, NVIDIA GeForce RTX 3080 GPU, and 12 GB RAM.

#### 2.2.3 Evaluation metrics

The performance of the trained model was assessed using three key metrics: Dice similarity coefficient (DSC), sensitivity (SEN), specificity (SPC), and intersection over union (IoU)^24^, ^25^, ^26^:

‐ DSC: The Dice coefficient (Equation 1) is a statistical metric used to gauge the similarity of two samples and the most common metrics for evaluating the performance of medical segmentation include pixel accuracy.
(1) $$DSC=\frac{2\times \left|X\cap Y\right|}{\left|X\right|+\left|Y\right|}$$
‐ IoU: Intersection over union value or Jaccard index (Equation 2) is defined by dividing the overlap between the segmented images using a predicted model and ground truth annotation by the union of these.
(2) $$IoU=\frac{X\cap Y}{X\cup Y}$$

where X and Y denote the ground truth and the segmented image obtained from the utilized DL models, respectively.

‐ SEN and SPC: Sensitivity (Equation 3) and specificity (Equation 4) quantify the overlapping ratio inside and outside the labeled volume. The sensitivity and specificity values were estimated using a confusion matrix to calculate: the sensitivity (true positive rate) and specificity (true negative rate) of the segmentation model.
(3) $$SEN=\frac{TP}{TP+FN}$$
(4) $$SPC=\frac{TN}{TP+FN}$$

here TP, FN, and TN refer to true positive, false negative, and true negative rates, respectively.

### 2.3 Impact of DL methods on the organ's S‐value

The workflow for the construction of the ground truth of a typical DL‐assisted internal dosimetry consists of DL‐based PET scan segmentation followed by the derivation of the S‐values through a set of MC simulations. The GEANT4 (GATE v8.1) simulation toolkit was performed for MC simulation and modeling. GATE was first developed for the simulation of medical imaging scanners^27^ and then was extended for radiotherapy and dosimetry applications.^28^ CT and PET images were imported as voxelized phantoms and voxelized sources, respectively. The half‐life (110 min) of the F‐18 tracer was also taken into account. The dose actor was used for scoring the voxel dose rate. The 3D S‐factor map was generated using a MATLAB script considering labeled CT images of the organs. The statistical uncertainties of the dose rates were estimated to be less than 0.1% at the voxel level.^29^, ^30^

To evaluate the impact of the segmentation method, the GATE MC simulator was employed. The two aforementioned DL approaches were compared with manual segmentation. The segmented PET images (either manually or DL‐based) served as a voxelized source in GATE. Similarly, the corresponding CT image was considered as the voxelized phantom to account for the anatomical details of each patient during the MC modeling. Some key details of MC simulation such as the physics model, dealing with voxelized sources and phantoms in GATE, and the scoring of the desired quantities are previously published by some of the authors.^29^ The S‐value for the bladder, liver, lungs, kidneys, stomach, heart's wall, and spleen were finally investigated and compared.

## 3 RESULTS AND DISCUSSION

### 3.1 Overall performance of 2D p2p‐U‐Net‐GAN and 3D HighRes3D_PET_

**FIGURE 6:**
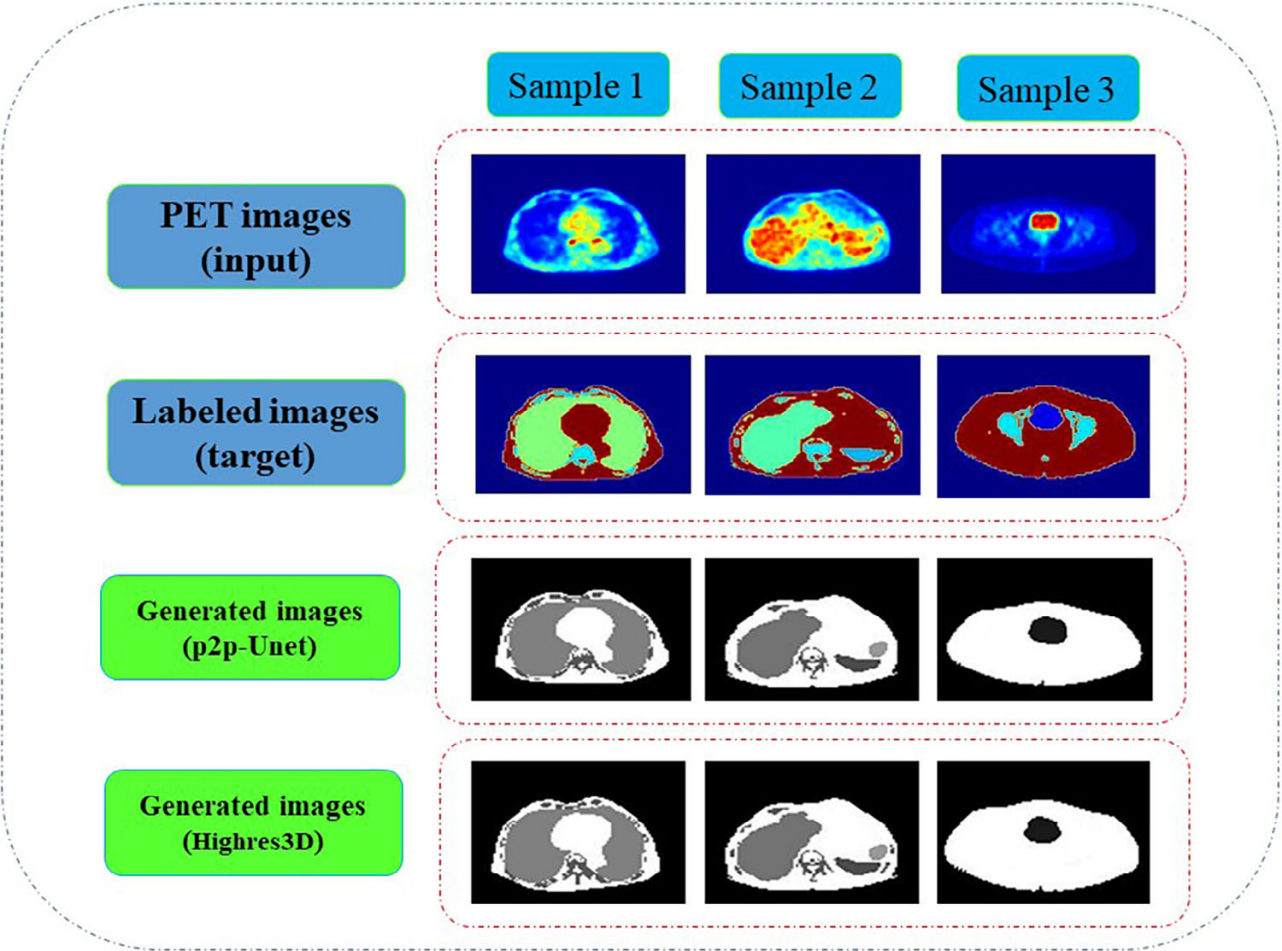
Representative segmentation results of one subject from the test dataset.

Figure 6 shows representative segmentation results of seven anatomical regions of the patient for the testing datasets using p2p‐U‐Net‐GAN and HighRes3D_PET_ architectures. The window size was kept the same for both training and testing. The p2p‐U‐Net‐GAN training losses for the generator, L1, discriminator, and GAN were estimated to be 0.99, 0.012, 0.5, and 0.8, respectively. The HighRes3D_PET_ loss was calculated to be 0.97, after 200 training epochs. The strategy for changing the learning rate of the models resulted in 2%−3% reductions in training losses compared to a constant learning rate and improved the performance of ROI segmentation. The entire training lasted approximately 3 h for HighRes3D_PET_ and 6 h for p2p‐U‐Net‐GAN.

### 3.2 Segmentation performances of the 2D p2p‐U‐Net‐GAN, 3D HighRes3D_PET_, HighRes3D_PET/CT_

The performance of the three models was assessed using the evaluation metrics listed in Table 2. Key evaluation metrics, such as SEN, SPC, and IoU were estimated (five patient subjects in the 3D model (the HighRes3D_PET_) and 1500 slices in the 2D model with separate testing datasets) and varied between 0.89–0.93, 0.98–0.99, and 0.87–0.89, respectively. An acceptable degree of similarity was observed between the generated and target images in the kidneys, lungs, liver, heart's wall, and spleen, whereas the bladder and axial skeleton regions showed a lower degree of similarity. As shown in Table 2, the evaluation metrics of the 3D models resulted in a congruous trend between the generated and target images. However, further similarity analysis revealed unmatched segmented areas and unacceptable evaluation metric values.

**TABLE 2:** Variation of evaluation metrics for 2D and 3D models.

Metrics
Methods	SEN (Test dataset)	SPC (Test dataset)	IoU (Test dataset)
p2p‐U‐Net‐GAN (2D)	0.89 ± 0.02	0.98 ± 0.002	0.87 ± 0.010
HighRes3D_PET_ (3D)	0.93 ± 0.02	0.99 ± 0.001	0.89 ± 0.011

**FIGURE 7:**
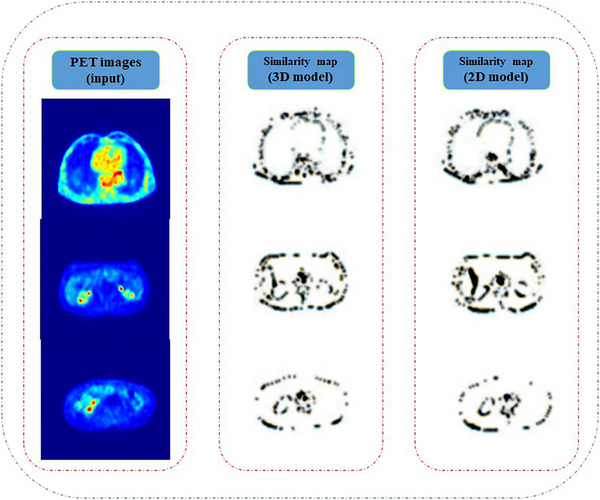
The difference map of p2p‐U‐Net‐GAN (2D) and HighRes3D_PET_ (3D) models.

Figure 7 shows difference maps of the ground truth and generated images for the three samples of the 2D and 3D models. The maps highlight the similar area of the two images and indicate that the vertebrae of the spine were not accurately segmented. The model is unable to generate the skeleton region using PET images as the input due to a lack of radiotracer uptake in bone structures.

**FIGURE 8:**
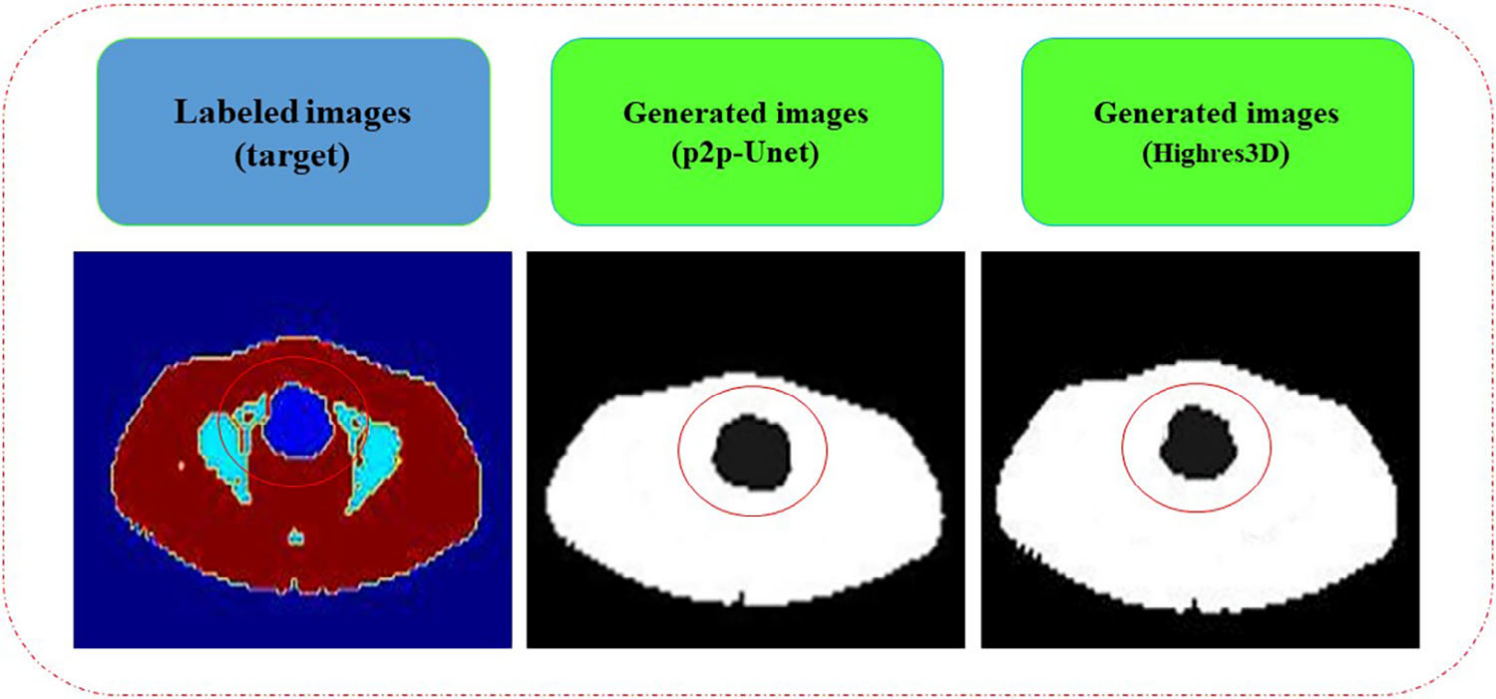
Representation of the inaccurate area of the bladder in generated slices.

Figure 8 shows the missed areas in the generated images, which were segmented and marked in the ground truth images. The bladder in each slice was overestimated owing to the aggregation of the 18F‐FDG radiotracer after injection. The reasons are^1^ considerable washout of radionuclide into balder at 60‐min post‐injection,^2^ filling the bladder with urine. Therefore, segmentation of the bladder using DL is challenging and inaccurate. The problem can be approached by either manual segmentation or other methods to avoid overestimation of its volume.

The mean Dice scores of the five subjects are listed in Table 3. It should be emphasized that there was an independent unseen testing dataset. HighRes3D_PET_ illustrates superior results than 2D p2p‐U‐Net‐GAN thanks to the pertaining with medical imaging data as NiftyNet is a medical imaging‐dedicated platform. Five anatomical regions including kidneys, lungs, liver, spleen, and body contour manifest high Dice scores (>0.90). The rest including the bladder and vertebrae of the spine, illustrate low Dice scores, particularly the skeleton region. As expected, HighRes3D_PET/CT_ offers superior performance in terms of Dice score compared to HighRes3D_PET_ mainly due to the inclusion of the extra anatomical CT data and additional features provided by the CT information. The difference between HighRes3D_PET_ and HighRes3D_PET/CT_ is more pronounced in the bladder as a difficult‐to‐segment organ in PET‐only images. Albeit, the performance of the HighRes3D_PET_ and HighRes3D_PET/CT_ is comparable indicating the robust application of HighRes3D_PET_ in the segmentation of PET images guided by anatomical CT data.

**TABLE 3:** Comparison of Dice scores of different organ regions for the p2p‐U‐Net‐GAN, HighRes3D_PET,_ and HighRes3D_PET/CT_.

Organs
Segmentation method	Liver	Spleen	Kidneys	Lungs	Stomach	Heart wall	Body region	Bladder	Skeleton region
HighRes3D_PET_	0.962	0.972	0.952	0.925	0.910	0.871	0.867	0.612	0.272
HighRes3D_PET/CT_	0.987	0.982	0.976	0.971	0.986	0.904	0.912	0.901	0.801
p2p‐U‐Net‐GAN	0.901	0.917	0.90	0.910	0.872	0.821	0.815	0.523	0.201

The evaluation metrics listed in Table 2 highlight the acceptable performance of both networks in several organ regions. However, the bladder and skeleton structures were segmented with low accuracy due to the lack of 18F‐FDG radiopharmaceutical uptake. The findings of the evaluation metrics, specifically the Dice score, show better accordance and similarity of generated images with the 3D networks and ground truth (see Tables 2 and 3). As mentioned in the Introduction, several reports have demonstrated the encouraging role of the DL methods in auto‐segmentation for different imaging modalities. Dong et al. reported the 0.75–0.97 dice scores for CT multiorgan segmentation^16^ and Bourigalt et al. obtained a mean Dice score of 0.75 for PET/CT tumor segmentation.^31^ Referring to our findings, the exploited 2D and 3D network architectures in this study result in acceptable Dice scores (0.82−0.96, except for the bladder and skeleton regions) indicating a promising performance in multiorgan segmentation of PET‐only images.

### 3.3 Correlation of manual and DL methods in estimating the organ volume

The low‐dose CT images were used for both manual segmentation (as a reference) as well as the preparation of the labeled dataset. Voxel‐based volume estimation for each organ. In a given slice, the volume of the organ is simply the multiplication of pixels representing the organ with slice thickness. The process is then repeated for all slices containing the organ of interest. Finally, the all‐calculated volumes in each slice were summed up. The volume of the organs was then estimated for ten test subjects using DL and manual methods and then compared with those of the manual segmentation. Table 4, lists the organ volume ranges estimated using three different methods. Referring to Table 4, the two investigated DL methods overestimate the volume of all investigated organs. Such an overestimation is more pronounced for the 2D p2p‐U‐Net‐GAN compared to the 3D architecture (i.e., the HighRes3D_PET_ network).

**TABLE 4:** Comparison of the mean estimated volume (in mL) of different organs using the p2p‐U‐Net‐GAN, HighRes3D_PET_, and manual segmentation methods.

Organs
Segmentation method	Liver	Spleen	Lungs	Kidney
p2p‐U‐Net‐GAN	1643 ± 320	260 ± 70	4772 ± 310	169 ± 20
HighRes3D_PET_	1640 ± 328	257 ± 78	4759 ± 370	166 ± 22
Manual	1638 ± 380	253 ± 78	4750 ± 360	162 ± 20

The volume of the spleen was then estimated for ten test subjects using both p2p‐U‐Net‐GAN and HighRes3D_PET_ methods (mean reference volume of spleen 253 mL, mean DL estimated volume of spleen 257 mL), and the repeatability and agreement of both were investigated using intraclass correlation coefficients (ICC) and Bland–Altman analysis.

**FIGURE 9:**
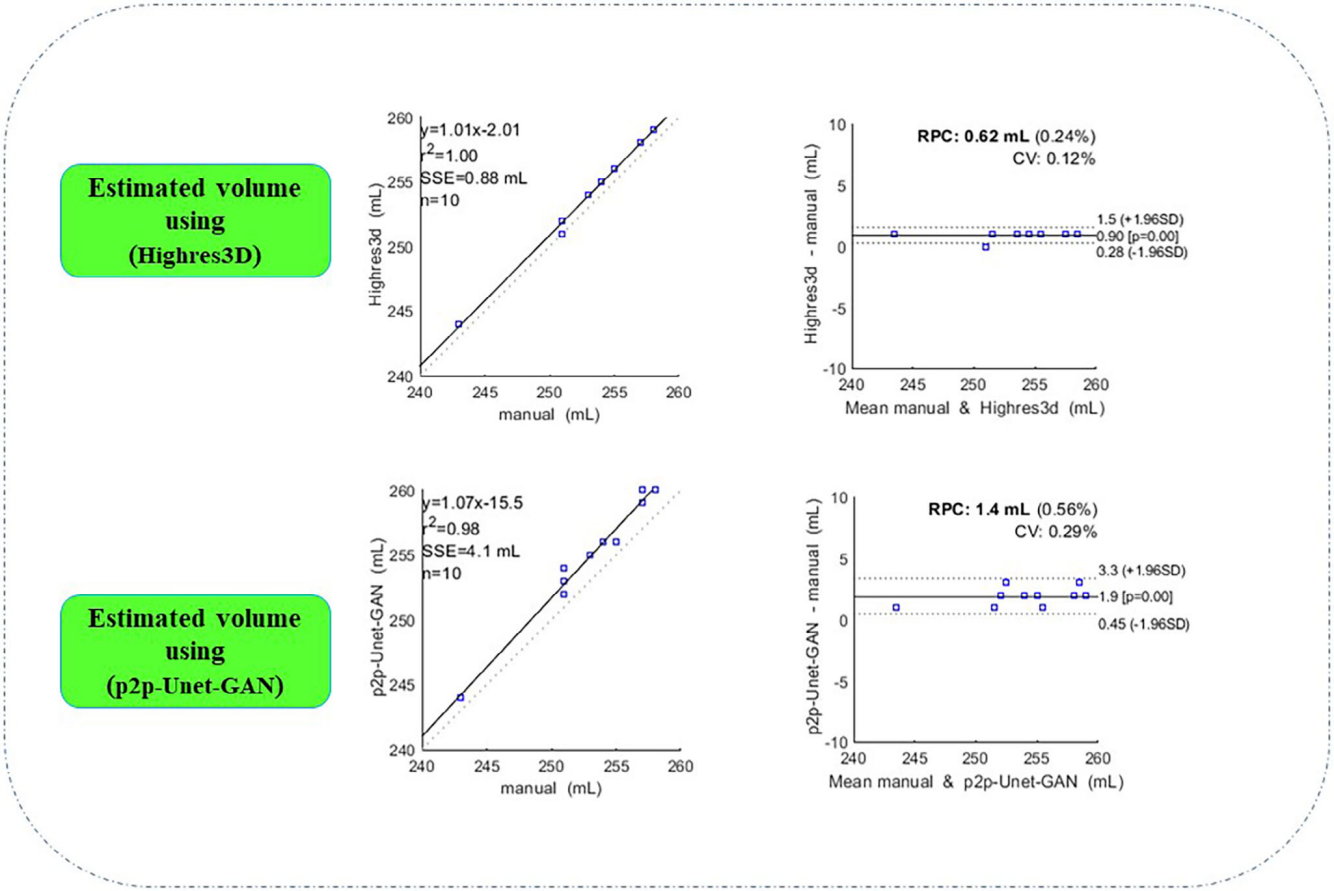
The Bland–Altman plots of spleen volume for the ten patients for the HighRes3D_PET_ and p2p‐U‐Net‐GAN architectures. The regression line has been also plotted in each graph.

The ICC between the two methods was moderate for the four organs (HighRes3D_PET_: 0.75 ± 0.05 and p2p‐U‐Net‐GAN: 0.71 ± 0.06). The spleen volume was calculated by counting nonzero elements multiplied by the X, Y, and Z spatial resolutions to obtain the real volume. Figure 9 shows the Bland–Altman plots and regression lines for estimated spleen volumes using HighRes3D_PET_ and p2p‐U‐Net‐GAN. The variance range for HighRes3D_PET_ was estimated to be in the range of 0.28–1.5 indicating a good correlation between the manual and DL‐assisted volume estimation segmentation methods. However, the p2p‐U‐Net‐GAN variance ranged from 0.45 to 3.3. The regression line reveals a meaningful correlation between the DL and manual segmentation methods with 0.88 and 4.1 mL of the sum of square error (SSE) values for HighRes3D_PET_ and p2p‐U‐Net‐GAN, respectively. In both DL approaches, the estimated spleen volume was higher than the manual estimation used as a reference, and the RPC values (return the coefficient of reproducibility) were 0.62 and 1.4 mL, respectively.

The Bland–Altman analysis showed acceptable results for both networks. The DL methods slightly overestimate the volume. The reasons can be explained by^1^ the partial volume effect in low‐resolution PET images and^2^ low‐dose and hence low‐quality CT‐based labels (ground truth). It should be highlighted that to reduce the radiation dose to the patients, acquiring low‐dose CT images is unavoidable. Surprisingly, the variance range of HighRes3D_PET_ volume estimation varied between 0.28 and 1.5 which was congruent with the manual segmentation. Overall, the NiftyNet platform exhibited superior performance compared to the p2p‐U‐Net‐GAN network in terms of accuracy due to its window sampling method and medical imaging‐specific features.

### 3.4 Comparison of the organ's S‐value between DL and manual segmentation methods

**FIGURE 10:**
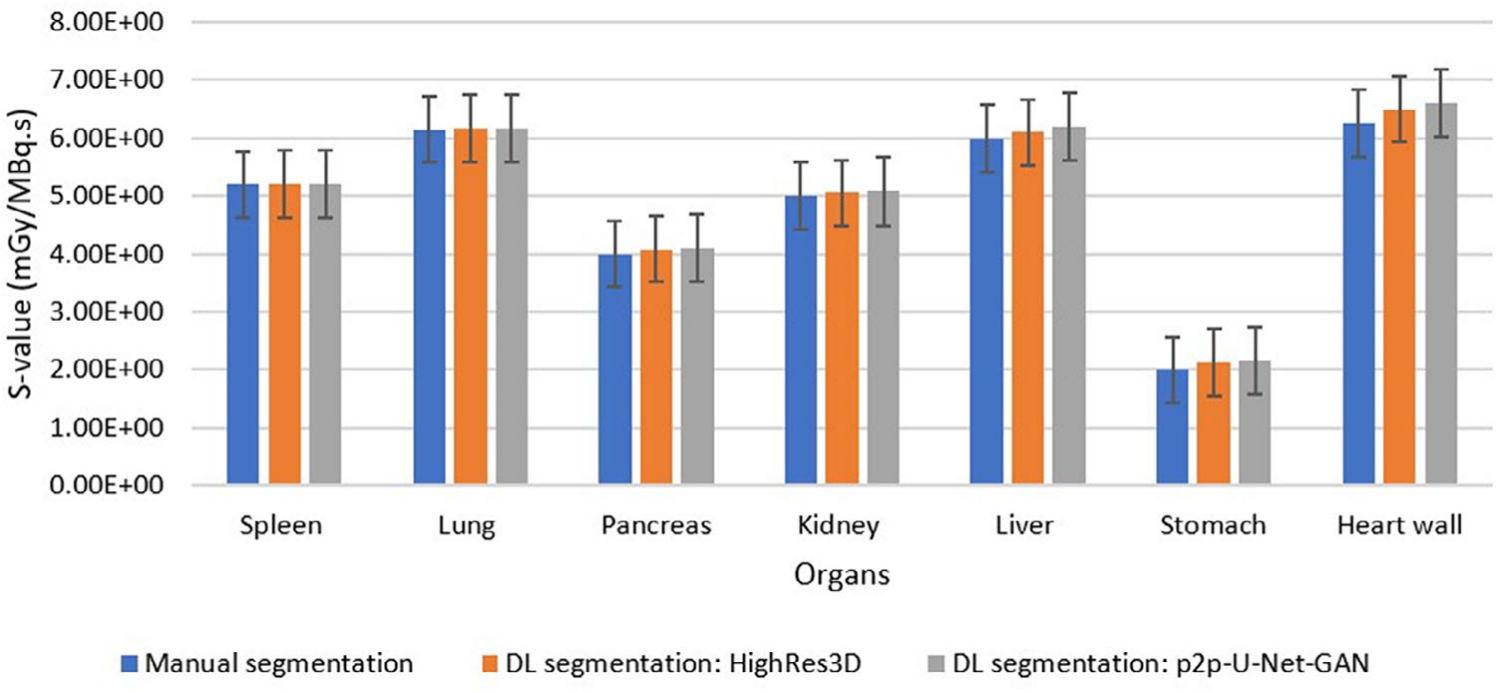
The comparison of the different organ's S‐values with manual and DL segmentation methods.

Figure 10 compares the organ's S‐value between the DL and manual segmentation methods. The percentage differences between the two methods varied between 0.1% and 6% with the maximum difference attributed to stomach doses. The evaluation metrics for stomach slices showed moderate values. Albeit, the two DL segmentation approaches show no significant difference in S‐value. The S‐values estimated by the DL segmentation were slightly overestimated. This is mainly due to the overestimation of the volume of the organs by the DL models. As expected, HighRes3D_PET_ offers superior performance in all organs of interest compared with the p2p‐U‐Net‐GAN in terms of accuracy mostly because of its capability to better predict the volume of organs. While the time consumption for the manual segmentation was around 4 h, the HigheRes3D_PET_ requires approximately 10 min to provide the output, all using the same PC.

It is worth noting that due to concerns arising from the radiation burden on the patients, in the present study, the ground truth (the labeled images) was based on the segmentation of low‐dose CT images. By enhancing the quality of low‐dose CT images towards that of the diagnostic ones utilizing another DL network, a superior‐quality ground truth would be obtained. Therefore, the performance of the investigated DL networks can be further improved by benefiting from higher‐quality ground truth.

## 4 CONCLUSION

In this work, we exploited p2p‐U‐Net‐GAN, as the first report on the topic, to segment PET images without incorporating prior anatomical data. The performance of the p2p‐U‐Net‐GAN was compared with that of the HighRes3D_PET_. The comparison of both networks demonstrates that the HighRes3D_PET_ provides superior performance mainly due to its inherent 3D nature and pretraining with medical images. Furthermore, the HighRes3D_PET_ better estimates the spleen volume compared with the p2p‐U‐Net‐GAN enabling internal dosimetry with higher accuracy. Both networks not only offer comparable results with manual segmentation but also with a much lower computational burden. While the incorporation of anatomical CT data in the segmentation of PET images (HighRes3D_PET/CT_) leads to a more accurate segmentation of organs of interest, particularly the bladder, the performance of the HighRes3D_PET_ remains comparable and encouraging. Therefore DL‐assisted segmentation can be considered the method of choice in commercial patient‐specific internal dosimetry software. The suggested DL segmentation methods can be generalized to non‐FDG PET as well as SPECT scans. The future work of the present study will focus on other vital organs such as the prostate, thyroid, and brain. Extending the application of the exploited DL networks to other imaging modalities such as functional magnetic resonance imaging (fMRI) will be an avenue for further research.

## AUTHOR CONTRIBUTIONS

Guarantor of integrity of the entire study: Sedigheh Sina. Study concepts and design: Mehrnoosh Karimipourfard, Sedigheh Sina, Mehran Yazdi. Literature research: Mehrnoosh Karimipourfard, Hojjat Mahani. Clinical studies: Mehrosadat Alavi, Mehrnoosh Karimipourfard. Experimental studies/data analysis: Mehrnoosh Karimipourfard, Hojjat Mahani. Statistical analysis: Mehrnoosh Karimipourfard, Sedigheh Sina, Hojjat Mahani, Mehran Yazdi. Manuscript preparation: Mehrnoosh Karimipourfard, Sedigheh Sina, Hojjat Mahani. Manuscript editing: Mehrnoosh Karimipourfard, Sedigheh Sina, Hojjat Mahani, Mehran Yazdi, Mehrosadat Alavi.

## CONFLICT OF INTEREST STATEMENT

The authors declare no conflict of interest.
